# Morphological diversity and ecological niche divergence in goitered and sand gazelles

**DOI:** 10.1002/ece3.6789

**Published:** 2020-09-19

**Authors:** Mahmoud‐Reza Hemami, Rasoul Khosravi, Colin Groves, Mohsen Ahmadi

**Affiliations:** ^1^ Department of Natural Resources Isfahan University of Technology Isfahan 8415683111 Iran; ^2^ Department of Natural Resources and Environmental Engineering, School of Agriculture Shiraz University Shiraz 7144165186 Iran; ^3^ School of Archaeology and Anthropology Australian National University Canberra ACT Australia

**Keywords:** cranial measurements, ecological niche modeling, goitered gazelle, sand gazelle, spatial niche overlap

## Abstract

The phylogeny and species boundaries of *Gazella subgutturosa* and *G. marica* have been long debated. The achievements of past conservation efforts have been compromised by a lack of knowledge about the phylogeny and taxonomic status of different populations. We integrated the recent genetic findings by previous studies with morphometric analyses and ecological niche modeling (ENM) to assess discreteness among populations of these gazelle species in Asia. Taxonomic diversity of gazelles was investigated by using principal components analysis (PCA) based on 14 cranial measures of male skulls. Ecological niche divergence was examined based on a PCA on climatic factors and a species distribution modeling (SDM) with environmental variables. Morphometric results indicated substantial differentiation in size between skulls of the western Zagros Mountains including west and south‐western Iran and Arabian Peninsula from all other samples east of the Zagros Mountains from Iran to China. ENM also revealed that gazelles in the east and west of Zagros Mountains occupy distinct niches and that there are apparent areas of disconnection across the goitered gazelle suitable range. A complete divergent niche occupation was also observed between goitered gazelles of northern Mongolia and other populations of the species, except those in China. Taking the inferences from ENM and morphology together with previous genetics results, we conclude that gazelles in the west and south‐west of Iran may represent *G. marica*. Also, our combined analyses revealed divergence among gazelles of Iran, Central Asia, and Mongolia/China. These results may pave the way for future studies and have conservation implications particularly for reintroduction/supplementation programs.

## Graphical abstract

The phylogeny and species boundaries of *Gazella subgutturosa* and *G. *marica have been long debated. We integrated the recent genetic findings by previous studies with morphometric analyses and ecological niche modeling (ENM) to assess discreteness among populations of these gazelle species in Asia. These results may pave the way for future studies and have conservation implications particularly for reintroduction/supplementation programs.

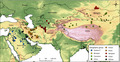

## 1 INTRODUCTION

Gazelles (genus *Gazella*) are widely distributed through mainly desert country from North Africa via the Middle East to Mongolia and India, although in most places, they have been depleted by overhunting. The *G. subgutturosa* is the most widespread species group in the genus *Gazella* (Groves & Grubb, 2011), ranging from Oman across the Arabian Peninsula to southern Turkey, and following the steppes of Central Asia eastwards into Central Mongolia, China, and Pakistan (Groves & Grubb, 2011). This wide geographic distribution encompassing diverse biogeographic zones has formed divergent populations in the species.

The taxonomy of sand gazelle (*G. marica*) and goitered gazelle (*G. Subgutturosa*) has been the subject of much debate. Sand gazelle was long considered to be a subspecies of *G. subgutturosa* on the basis of morphology and karyology (Groves & Harrison, 1967; Kingswood, Rebholz, Vassart, & Kumamoto, 1997; Kingswood, Vassart, & Williamson, 1996). Thomas (1897) described the Arabian sand gazelle as a distinct species, *G. marica* (Thomas, 1897); Ellerman and Morrison‐Scott (1951) considered it as a subspecies of the Saharan *G. leptoceros* (Cuvier, 1842)*;* and it was later suggested by Groves and Harrison (1967) that it is associated more closely with *G. subgutturosa*, probably as a subspecies of it. Wacher et al. (2011) provided a phylogenetic framework based on the analysis of mtDNA sequences of a number of wild and captive individuals throughout the species' natural range and restored *G. marica* to full species which is distributed in Arabian Peninsula, Iraq, Jordan, Syria, and Turkey. Other authors (Durmuş, 2012; Groves & Harrison, 1967; Kingswood & Kumamoto, 1988; Mallon & Kingswood, 2001) indicated that gazelles in a broad geographic area between Euphrates‐Tigris basin and the Zagros Mountains of Iran have morphologically mix characters. More information on the taxonomy of goitered gazelle is provided by Lerp et al. (2016), Abduriyim, Zibibulla, Eli, Ismayil, and Halik (2018), Silva (2019), and Fadakar et al. (2020).

There is a paucity of data on the phylogenetic status of gazelle populations in the northern geographic range of *G. marica*. Fadakar et al. (2019) found mitochondrial haplotypes of *G. marica* in south‐western Iran. They attributed their finding to either hybridization between *G. subgutturosa* and *G. marica* in their contact zone, or the existence of *G. marica* populations in Iran. Accordingly, the dunes and desert areas of south‐west and west of Iran at the border of Iran and Iraq are probably the historical habitat of sand gazelles. The resemblance of gazelles in south‐western Iran to *G. marica* has hitherto been noted by Groves and Harrison (1967). They described the size and morphology of gazelles from south‐western Iran as an intermediation between *G. subgutturosa* and *G. marica*. Groves (1997) suggested that gazelles in eastern Iraq and south‐western Iran have probably been arisen through secondary intergradation between *G. s. marica* and *G. s. subgutturosa*.

Groves and Grubb (2011) recognized four phylogenetic species for *G. subgutturosa* group including *G. subgutturosa*, *G. gracilicornis*, *G. yarkandensis*, and *G. marica*. Subsequent studies suggested the elevation of the latter to full species (Wacher et al., 2011), but the three others are generally considered as subspecies of *G. subgutturosa* (Abduriyim et al., 2018; Sorokin, Soldatova, Lukarevskiy, & Kholodova, 2011). However, based on mitochondrial genes, Fadakar et al. (2020) suggested that *G. s. gracilicornis* is synonymous with *G. s. yarkandensis*. Fadakar et al. (2020) recognized Lut and Kavir deserts in Central Iran as the main geographic barriers between western (Central Iranian; *G. s. subgutturosa*) and eastern (Asiatic; *G. s. yarkandensis*) populations of goitered gazelle and suggested that the distribution of *G. s. subgutturosa* extends to the west of the Zagros Mountains.

Another case of taxonomic debate about gazelles in south‐western Iran is the status of *G. karamii*, a taxon initially described by Groves (1969) as a subspecies of *G. bennettii* (Sykes, 1831) from Borazjan, south‐western Iran near the Persian Gulf. The specimen was later transferred to the *G. gazella* group, mainly due to its dark pelage (Groves & Grubb, 2011). However, based on morphometric analysis, Bärmann et al. (2013) found that the type of *G. karamii* was close to *G. marica*, and they suggested that it was possibly a synonym of it. Until now, no molecular data exist for the only specimen of *G. karamii* preserved in the MfN Berlin museum (ZMB MAM 41400). There has also been taxonomic uncertainty in gazelles of Khark (or Kharg) Island in the south of Iran. Based on cranial and morphometric analyses, Karami, Hemami, and Groves (2002) suggested that Khark Island population along with those in the west of the Zagros Mountains in Bushehr and Khuzistan provinces should be considered as a distinct subspecies of *G. subgutturosa*. Later, Mirzakhah, Naderi, Rezaei, Fadakar, and Naseri (2015) indicated that there is no evidence of *G. marica* haplotypes in this population.

Effective biodiversity conservation requires knowledge of ecological and phenotypic variation among taxa and their evolutionary relationships (Noguerales, Cordero, & Ortego, 2018). For many gazelle taxa, the achievements of past conservation efforts have been compromised by a lack of knowledge and confusion about the phylogeny and taxonomic status of different populations, and even species boundaries have not been certain (Groves & Grubb, 2011). Combining multiple sources of data, including genetic, ecological, and phenotypic data, can provide greater insight into the species boundaries (Ahmadi et al., 2018; Leaché et al., 2009). In this regard Lerp et al. (2016), Silva et al. (2017), and Fadakar et al. (2020) suggested the use of morphological and nuclear genetic studies to investigate further the relatedness of gazelle populations on the two sides of the Zagros Mountains.

We used phenotypic (cranial) and ecological (niche) data to examine whether gazelle populations on the two sides of the Zagros Mountains exhibit distinct phenotypic and ecological traits. Our study also includes skull measurements and occurrence locations collected across the entire range of *G. subgutturosa*, and accordingly, can assist in better understanding of the continent‐wide intraspecific variations among the species populations.

## 2 MATERIAL AND METHODS

### 2.1 Species concept

Since the 1990s, there has been much discussion about “what is a species”; de Queiroz (2007) cogently argued that a species is an evolutionary lineage, and that traits such as reproductive isolation, specific mate recognition systems, an ecological niche, increased genetic differentiation, and subjective “enough difference,” which have often in the past been used actually to define a species, develop along the lineage over time; the initial marker of the individuated lineage that is a species is likely to be diagnosability. In this paper, we follow de Queiroz's unified concept of species defined as “separately evolving metapopulation‐level lineages” (de Queiroz, 2007).

Based on morphometric analyses and as suggested by Wacher et al. (2011), we considered *G. marica* a distinct species from *G. subgutturosa*. We followed Fadakar et al. (2020) for the intraspecific classification of *G. subgutturosa*.

### 2.2 Data collection

**FIGURE 1:**
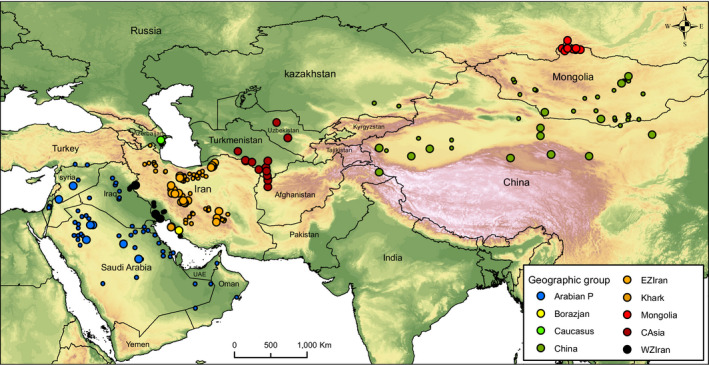
Locations where gazelle skulls (large colored circles) and presence points of *Gazella subgutturosa* and *G. marica* (small colored circles) were collected. See Table S1 and Figure S1 for geographic names and locality abbreviations, and gazelle skulls number respectively

A total of 138 skulls were measured. Skull specimens with more than one missing measurement were excluded from further analysis and 74 skulls collected from Arabian Peninsula (hereafter Arabian P), west of the Zagros Mountains in Iran and Iraq (hereafter WZIran), Khark Island in the Persian Gulf (hereafter Khark), east of Zagros Mountains in Iran (hereafter EZIran), Caucasus, Central Asia (north‐east Iran, Turkmenistan, Uzbekistan; hereafter CAsia), Mongolia, China, and Borazjan district (South‐western Iran on the mainland, opposite Khark island; hereafter Borazjan) were used to investigate the morphological diversity of the goitered gazelle group across its distribution range in Asia (Figure 1). Only adult male skulls were included in the study as not enough female skulls were available. Adult specimens were recognized by the complete eruption and full occlusion of premolars and molars (Angelici & Luiselli, 1999). Specimens from across the range of the group were studied between 1980 and 2010 in several museums and institutions. The measurements were taken by C. P. Groves and M. R. Hemami. Specimens kept in the Iranian National Museum of Natural History (MMTT) were measured by both authors; the difference in measurements was <0.2 mm. The list of skull specimens and their numbers in PCA plots is presented in Table S1 and Figure S1 in Online Resource 1. For cranial evaluation, fourteen linear skull parameters were chosen (Table S2; Figure S2). Mandibular measurements were excluded because the mandible was missing in many skulls. All measurements were taken with vernier callipers to the nearest 0.1 mm.

### 2.3 Data transformation

Because multivariate analyses are sensitive to missing data (Daneri, Esponda, De Santis, & Pla, 2005), specimens were excluded if data were missing for more than one measurement. To estimate the missing value for each specimen with only one missing data point, we used stepwise regression from the remaining subset of cranial variables available for that specimen (Reig, 1992; Reig & Ruprecht, 1989). In order to separate the effects of size and shape on morphology, we followed the approach of Jungers, Falsetti, and Wall (1995) and Klein, Franciscus, and Steele (2010). We used three derived variables from the original raw measurements to investigate the craniometric differences among specimens: (a) log_10_ of each measurement on each specimen was taken to allow for normal distribution and homogeneity of variances (log size and shape variables; Lewontin, 1966). (b) To obtain an index for overall size (Darroch & Mosimann, 1985; Mosimann & James, 1979), the geometric mean of all cranial dimensions for each individual was calculated (Klein et al., 2010). (c) The geometric mean of the specimen was subtracted from logged measurements. This difference is a measure of log‐shape and ratios between each raw measurement and overall size or geometric mean. In log‐shape variables, the variance is partitioned due to the specimen's size and shape; therefore, they are more readily justified than arbitrarily selected ratios between untransformed measurements for multivariate analysis (Klein et al., 2010). Correlation coefficients between transformed variables and greatest skull length were calculated to check if the data transformation was effective in removing the effect of size (Khosravi, Kaboli, Imani, & Nourani, 2012).

### 2.4 Data analysis

To identify the combination of variables that best separate skull samples and to define morphologically similar population groups, a principal component analysis (PCA) was performed applying both log size and shape and log‐shape variables. We calculated the correlation coefficient between cranial scores and the geometric means for log size and shape variables and log‐shape variables in the first four components. The relationship between size and shape was inspected by calculating the correlation between each of the log‐shape measurements and the geometric mean for all samples. Finally given the geographic distribution of the specimens, we assigned them into nine groups and projected observations position on two first PCs using the geographic prior. These geographic groups include Arabian P, WZIran, EZIran, Khark, Borazjan, Caucasus, CAsia, China, and Mongolia. Assigning specimens to the nine groups was done regarding the biogeographic structuring, geographic morphology of the region, and recent findings of Fadakar et al. (2020). We performed PCA and the associated graphs in R using “ade4” and “ggplot2” packages, respectively.

### 2.5 Ecological niche modeling procedure

To examine ecological divergence between gazelle groups, we adopted a between‐taxa species distribution modeling (SDM) based on environmental variables and a between‐group niche comparison based on a PCA analysis on climatic variables. For the first procedure, we conducted a maximum entropy (MaxEnt) model to predict highly suitable habitats for the three taxa of *G. marica* (Wacher et al., 2011), *G. s. subgutturosa*, and *G. s. yarkandensis* (Fadakar et al., 2020). To do so, four categories of environmental variables including land cover, anthropogenic, topographic, and climatic were compiled with occurrence points to conduct gazelles habitat suitability model. First, a total of 280 presence points were obtained from a variety of sources including direct field surveys from 2014 to 2018 by the authors, museum‐based collections, extraction from Global Biodiversity Information Facility (GBIF), and scientific literature review (Table S3). For those with no coordinates but exact locality names, records were georeferenced using the global gazetteer version 2.3 (http://www.fallingrain.com/world). The reliability of all records was assessed by mapping them in Google Earth version 7.1. We did not include gazelle populations in the contact zone of *G. subgutturosa* and *G. marica* in western Iran and Iraq as either we did not have skull specimen from them, or their genetic status was unknown. Moreover, we excluded Khark population and Borazjan gazelle from the ENM analysis, as the origin (native or introduced) of gazelles in Khark, and the locality of Borazjan gazelle was unknown.

To prepare environmental variables for MaxEnt analysis, we extracted four land cover types of the mosaic of herbaceous with sparse tree and shrub, sparse herbaceous, consolidated land (e.g., hardpans, gravels, and rocks), and unconsolidated land (e.g., bare soils and shifting sands) from the Global Land Cover by National Mapping Organization (GLCNMO) version 3 (Kobayashi et al., 2017). We then calculated the proportion of each cover type in a 7 × 7 grid cell moving window neighborhood analysis. To incorporate anthropogenic impact, we used human footprint as a measure of human influence on the land surface based on the data derived from the 2009 Human Footprint (Venter et al., 2018). Using the Shuttle Radar Topography Mission (SRTM) elevation model (http://srtm.csi.cgiar.org), the two most important topographic variables were compiled: elevation and topographic roughness, that is, standard deviation (*SD*) of the elevation of all raster cells included in the 7 × 7 grid cells. Three climatic variables, including annual mean temperature, temperature seasonality, and annual precipitation, were obtained from WorldClim dataset (Hijmans, Cameron, Parra, Jones, & Jarvis, 2005). Modeling was repeated 10 times based on a cross‐validation method, and the predictive performance of models was evaluated based on the area under the receiver operating characteristic curve (AUC). The pairwise spatial overlap between derived habitat suitability models was then calculated based on Schoener's *D* niche overlap using ENMTools (Warren, Glor, & Turelli, 2010). Schoener's D ranges from 0 to 1 and represents a gradient of complete dissimilarity to fully overlapping niches.

For between‐group niche comparison, we combined species presence points of all the gazelle groups, extracted 19 climatic variables of the WorldClim dataset, calculated the orthogonal climatic axes, and depicted the position of the species occurrence points and habitat background as a representative of their ecological niche on climatic axes. This method, similar to the PCA on morphometric traits, merges all the observation in a pool and perform the analysis based on the whole dataset. To quantify niche divergence between gazelles groups, we used PCA‐env method proposed by Broennimann et al. (2012) in R environment. As the first step of the PCA‐env analysis, the density of occurrences and environmental variables using a kernel density function (*R* = 100) was calculated in the multivariate PCA space. Next, the observed niche overlap score of the seven gazelle groups across the gradients of the PCA space was computed based on a Schoener's *D* metric. Finally, two randomization tests called “niche equivalency” and “niche similarity” were used to test the hypotheses of niche divergence. The available niche space for the gazelle groups was defined as all pixels of the 19 climatic variables within a buffer of 50‐km enclosing the species presence points.

## 3 RESULTS

### 3.1 Morphometric analysis

The number of specimens for each location and some descriptive statistics of 14 skull measurements are presented in Table S4.

#### 3.1.1 Principal components analysis based on log size and shape variables

**FIGURE 2:**
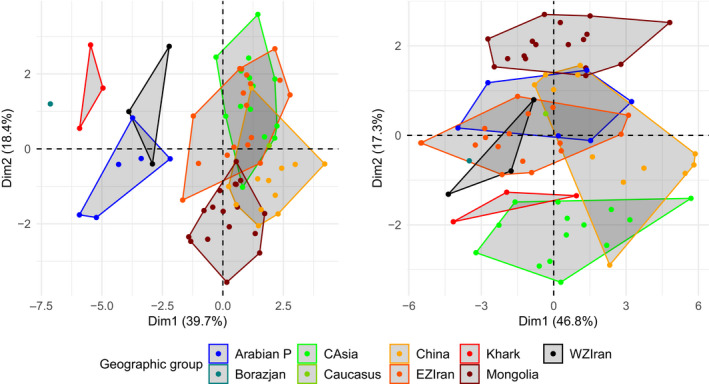
The plot for the 14 cranial scores of ungrouped gazelle populations on the first and second components based on log size and shape (left) and log‐shape measures (right). Arabian P: Arabian Peninsula, WZIran: west of the Zagros Mountains in Iran and Iraq, EZIran: east of Zagros Mountains in Iran, Khark: Khark Island, Borazjan: south‐western Iran on the mainland, opposite Khark Island, CAsia: north‐east Iran, Turkmenistan, and Uzbekistan

In Table 1, the eigenvalues for the first four principal components and contribution of 14 log size and shape cranial measurements are presented. The first and second axes of the PCA accounted for 39.8% and 18.4% of morphological variations, caused by log size and shape variables, respectively. All variables contributed positively to the PCA loading factors. Skull length, biorbital breadth, and breadth of braincase were most highly correlated to PC1, and greatest width across horns, horn length, and distance between tips of horns were most highly correlated to PC2. We address only the first two components because these components contain most of the information on how well log size and shape variables separate skulls from different regions. Figure 2 plots the first and second principal component scores against each other; those groups that do not overlap on PC1 differ significantly in size (mainly of the skull), while those that do not overlap on PC2 differ significantly in shape (especially of the horns). Figure 2 shows that size and shape, singly or together, distinguish skulls from Arabian P, WZIran, and Khark from all other samples. The type of *G. karamii* is (i.e., Borazjan) located close to the sample from Khark. All gazelle populations on the east of the Zagros Mountains from Iran to China show some degree of overlap. However, those in EZIran and CAsia are highly differentiated from the Mongolian populations.

**TABLE 1:** Summary of the two separate principal component analyses performed on the 14 craniometric variables of gazelle populations

Component	Log size and shape variables				Log‐shape variables			
	1	2	3	4	1	2	3	4
Eigenvalue	5.566	2.576	1.626	1.279	6.56	2.43	1.79	0.98
Percent	39.76	18.40	11.61	9.13	46.84	17.36	12.80	6.99
Cumulative percent	39.76	58.16	69.77	78.90	46.84	64.20	77.00	83.99
Contribution of the variables								
HL	1.20	20.66	8.49	2.65	0.12	16.63	17.00	6.09
DBTH	0.42	19.88	0.23	26.60	9.38	5.60	7.34	0.59
GWH	0.71	23.30	1.40	17.86	5.36	15.49	6.46	1.41
OHW	9.06	3.70	15.36	7.19	7.21	16.28	3.57	0.63
BNA	7.90	6.37	0.10	8.22	8.13	3.42	3.23	3.21
BNP	6.12	9.14	11.13	10.23	6.06	2.66	20.77	6.23
NL	9.20	1.17	1.55	6.93	1.83	5.92	7.83	56.88
SL	15.16	0.04	3.32	0.03	12.61	0.00	0.24	6.68
BB	14.14	0.00	0.45	0.23	12.30	0.95	0.72	2.27
PL	0.78	2.64	45.44	8.37	1.76	23.34	16.53	0.07
MTRL	10.92	0.01	0.65	2.25	10.05	1.53	0.15	1.15
PB	6.60	4.36	3.45	0.61	9.02	0.57	2.54	14.47
BBC	12.03	0.25	0.63	0.33	10.50	0.18	0.06	0.32
BL	5.77	8.48	7.81	8.51	5.66	7.42	13.56	0.01

Note

Two separate PCAs were performed based on log size and shape variables and log‐shape variables.

#### 3.1.2 Principal components analysis based on log‐shape

In Table 1, the eigenvalues for the first four principal components and contribution of 14 log‐ shape variables are presented. The first and second axes of the PCA respectively accounted for 46.8% and 17.4% of the total variance. All variables contributed positively to the PCA loading factors. Again, skull length, biorbital breadth, and breadth of braincase were most highly correlated to PC1, and horn length, outer horn base width, and greatest width across horns were most highly correlated to PC2. Figure 2 plots the first and second principal component scores against each other. There is a clear separation between samples from CAsia, Mongolia, and EZIran, implying that skull specimens obtained from these regions are rather differentiated based on shape than size. In contrast to log size and shape variables, there is considerable overlap between samples from EZIran, WZIran, and the Arabian P. As gazelle populations in west and east of the Zagros Mountains are overlapped in log‐shape variables, the type of *G. karamii* (Borazjan) is thus located close to both groups.

#### 3.1.3 The interaction of size and shape

The results of the correlation between the principal component scores and the geometric means for log size and shape variables in the four first components showed that there is a significant correlation between the first component and the geometric mean (Table 2). Since in PCA, the first component reflects the size, it was expected that the correlation between component 1 and the geometric mean would be tight (Figure S3). The correlation between the scores of the samples based on cranial variables and the second component was low and insignificant (Figure S3; Table 2). Correlation between scores of individuals' log‐shape variables and the geometric mean showed that no components were correlated significantly with size (the geometric mean), implying that skull size has no impact on skull shape (Jungers et al., 1995; Klein et al., 2010).

**TABLE 2:** Pearson's *r* correlation coefficients between principal component scores and the geometric means of each variable

Component	Log size and shape	Log‐shape
1	**−0.458**	−0.115
2	0.068	−0.121
3	0.19	0.167
4	**0.236**	0.22

Note

Bold‐face marks show coefficients that are significant at the 0.05 level or below.

Table 3 presents the correlation coefficients between the individual log‐shape variables and corresponding geometric means. A coefficient near 0 indicates isometry, a positive coefficient indicates positive allometry, and a negative coefficient indicates negative allometry (Klein et al., 2010; Mosimann & James, 1979). The measured variables tend to increase more slowly than the overall size (Table 3). Horn variables (DBTH and GWH) showed a significant positive correlation, but HL also showed a non‐significant negative correlation, meaning that horn variables tend to increase more rapidly than the overall size. Skull length showed a significant negative correlation and appears to increase particularly slowly.

**TABLE 3:** Pearson's *r* correlation coefficients between the log‐shape variables and the geometric means

Variable	Log‐shape	Variable	Log‐shape
HL	−0.135	SL	**−0.572**
DBTH	**0.583**	BB	**−0.784**
GWH	**0.352**	PL	0.212
OHW	**−0.440**	MTRL	**−0.571**
BNA	**−0.514**	PB	**−0.651**
BNP	**−0.290**	BB	**−0.582**
NL	0.070	BL	**−0.669**

Note

Bold‐face marks show coefficients that are significant at the 0.05 level or below

### 3.2 Ecological niche divergence

**FIGURE 3:**
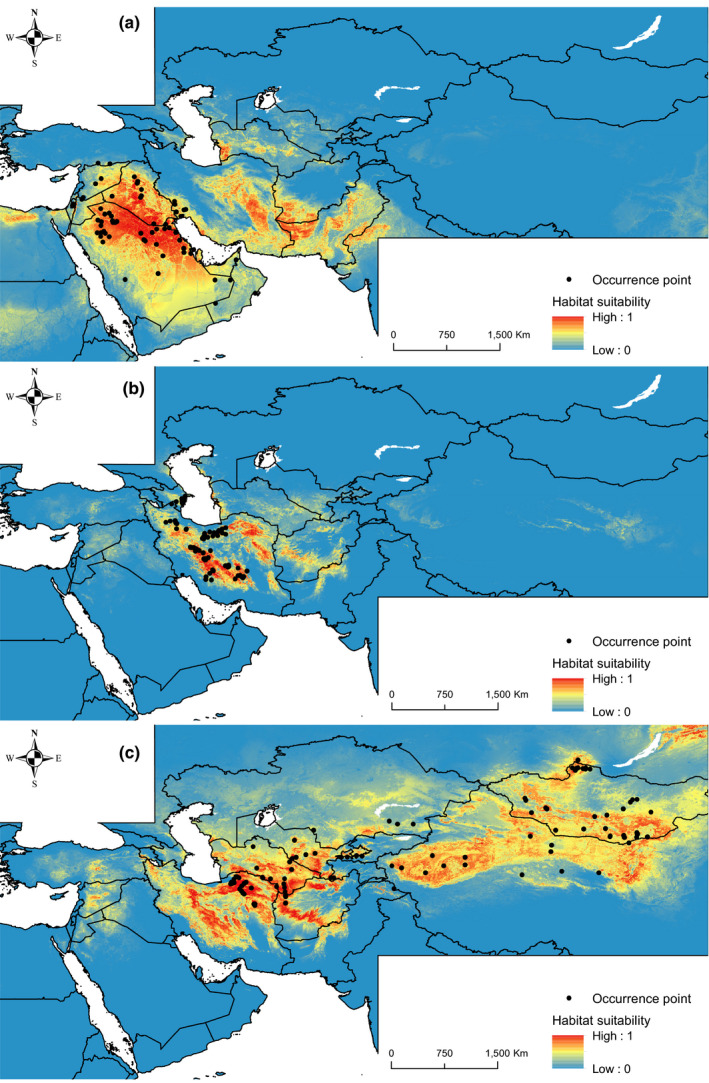
Predicted habitat suitability of *Gazella marica* (a), *G. s. subgutturosa* (b), and *G. s. yarkandensis* (c) based on the MaxEnt model

**FIGURE 4:**
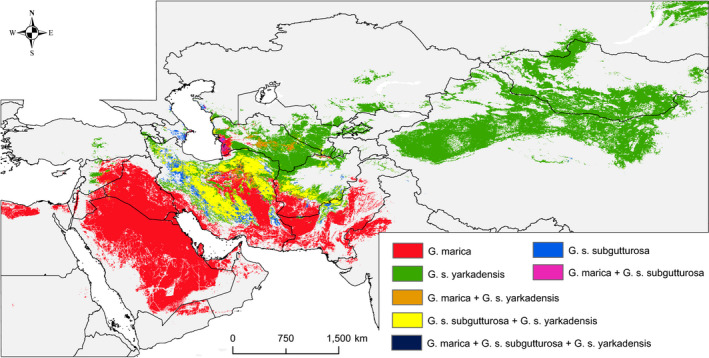
Spatial overlap in the suitable habitats of *Gazella marica*, *G. s. subgutturosa*, and *G. s. yarkandensis*. Suitable habitats were identified based on the minimum 10 percentile of habitat suitability at the presence points of each species as the presence/absence threshold

Notable consistency was detected in the predicted habitat suitability of gazelles when compared with occurrence records (Figure 3). Accordingly, the mean AUC of 0.914, 0.922, and 0.900 was obtained for the MaxEnt model of *G. marica*, *G. s. subgutturosa*, and *G. s. yarkandensis*, respectively. For *G. marica* annual mean temperature, unconsolidated land, temperature seasonality, and consolidated land, for *G. s. subgutturosa* annual mean temperature, roughness, human footprint, and for *G. s. yarkandensis* annual mean temperature, sparse herbaceous, altitude, and temperature seasonality were the most important variables contributing to the species habitat suitability model. A relatively high magnitude of spatial overlap was seen in the habitat suitability of *G. s. subgutturosa* and *G. s. yarkandensis* where a Schoener's *D* overlap score of 0.34 was obtained. Pairwise spatial overlap of *G. marica*–*G. s. subgutturosa* and *G. marica*–*G. s. yarkandensis* was 0.17 and 0.12, respectively (Figure 4). A noteworthy habitat disconnection was seen in habitat suitability of the *G. s. yarkandensis* where suitable ranges of central Asia were separated from China and Mongolia. Considering response curves of the environmental variables, we found that *G. marica* prefers lower‐elevated habitats mostly covered by unconsolidated lands with higher temperature and lower temperature seasonality compared to *G. s. subgutturosa* and *G. s. yarkandensis* (Figure S4).

**FIGURE 5:**
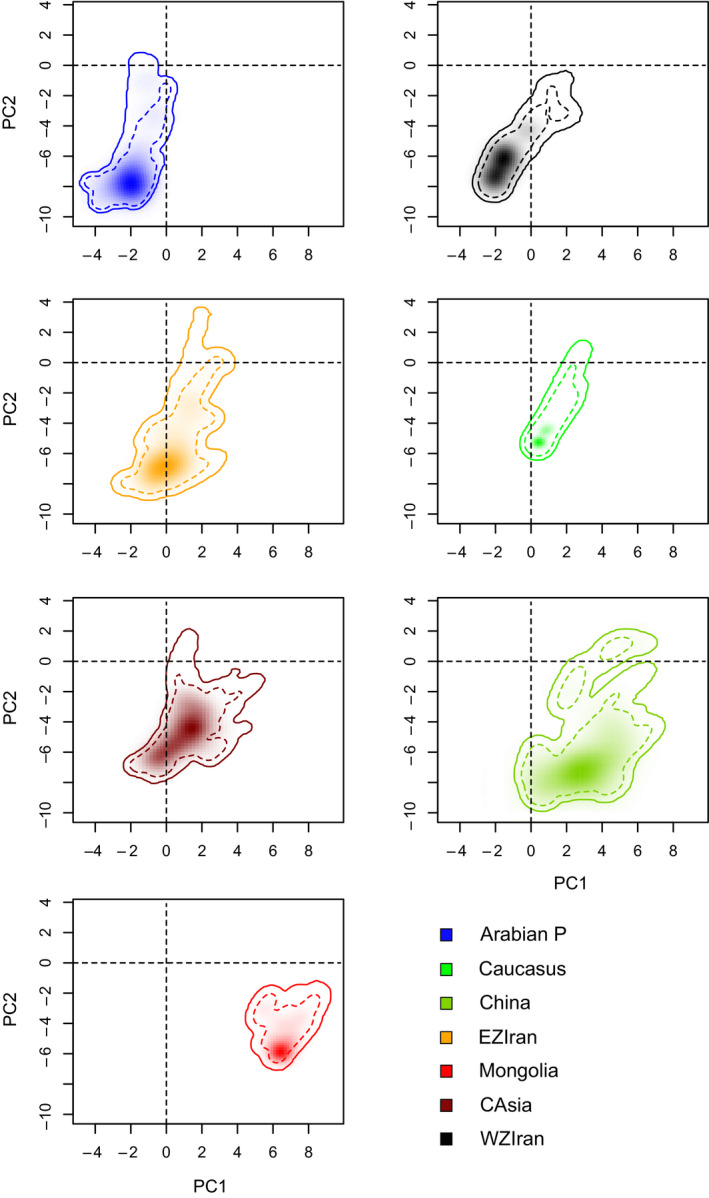
The ecological niche of the gazelle groups along multivariate climatic axes derived from PCA‐env method. Transparent shading indicates density of the presence points of each gazelle group per cell, and dashed and solid contour lines show 50% and 100% of the background environmental space, respectively

For between‐group niche comparisons, PC1 and PC2 of the climatic variables explained 49.6% and 20.8% of the variation in climatic variables, respectively. PC1 was mostly correlated with mean variables of temperature and precipitation while PC2 was highly correlated with standard deviation of temperature (temperature seasonality; Table S5). Pairwise comparisons of climatic niches along the PC1 and PC2 of the PCA‐env showed low between‐group and comparably higher within‐group niche overlap values for western Zagros (WZ; i.e., Arabian P and WZIran) and eastern Zagros gazelle groups (i.e., EZIran, CAsia, Caucasus, China, and Mongolia). The highest niche overlap values were obtained for EZIran–CAsia (*D* = 0.26) and Arabian P–WZIran (*D* = 0.23), while both Arabian P and WZIran showed zero or very low niche overlap with all the eastern gazelle groups, except for EZIran group (Table 4). Considering niche position on climatic axes (Figure 5), China and EZIran groups occupied largest and the Caucasus occupied narrowest climatic niche.

**TABLE 4:** Pairwise niche overlap scores of the gazelle groups in terms of Schoener's *D*

	Niche Overlap	Equivalency (*p*)	Similarity 1 to >2	Similarity 2 to >1
Arabian P–WZIran	0.23	**<.01**	**0.02**	0.435
Arabian P–EZIran	0.10	**<.01**	**0.02**	**0.02**
Arabian P–Caucasus	0	—	—	—
Arabian P–CAsia	0.07	**<.01**	**0.02**	**0.02**
Arabian P–China	0	—	—	—
Arabian P–Mongolia	0	—	—	—
WZIran–EZIran	0.09	**<.01**	**0.02**	**0.02**
WZIran–Caucasus	0.04	**<.01**	**0.02**	**0.02**
WZIran–CAsia	0.06	**<.01**	**0.02**	**0.02**
WZIran–China	0	—	—	—
WZIran–Mongolia	0	—	—	—
EZIran–Caucasus	0.10	**<.01**	0.97	0.93
EZIran–CAsia	0.26	**<.01**	0.57	0.69
EZIran–China	0.11	**<.01**	0.26	0.35
EZIran–Mongolia	0	—	—	—
Caucasus–CAsia	0.09	**<.01**	0.21	0.25
Caucasus–China	0.02	**<.01**	**0.02**	**0.02**
Caucasus–Mongolia	0	—	—	—
CAsia–China	0.10	**<.01**	0.45	0.26
CAsia–Mongolia	0	—	—	—
China–Mongolia	0.08	**<.01**	0.18	**0.05**

Note

Niche equivalency *p*‐values and niche similarity *p*‐values obtained from a randomization test. Bold values are showing significant values with *p* < .05 in the niche equivalency test and falling outside the 95% confidence interval of the niche similarity test.

The niche equivalency hypothesis was rejected for all pairwise comparisons, showing that niches of all gazelle groups are significantly distinct (niche equivalency test: *p < *.01; see Table 4). Background similarity test also showed a significant niche difference between WZIran and EZIran. Arabian P–WZIran niche comparison indicated that there is a significant difference (*p* < .05) in niche overlap between WZIran observed niche and niche selected randomly from the Arabian P. For pairwise comparisons of EZIran with other eastern gazelles (Caucasus, CAsia, China), we found no significant niche differences. While niche comparison of the Caucasus‐China was significantly different, we found no significant background difference in the Caucasus–CAsia and CAsia–China niche comparisons (Table 4). Results also showed a complete divergent niche occupation (*D* = 0) between gazelles of Mongolia and other Asiatic gazelles, except for the China–Mongolia niche comparison for which niche overlap was very low (*D* = 0.08) and their background niche was significantly different (*p* < .05; Figure 5 and Table 4).

## 4 DISCUSSION

### 4.1 Morphometric analysis

In recent years, both the genetic results of Wacher et al. (2011) and the craniometric measurements of Groves and Grubb (2011) revealed a strong separation between *G. subgutturosa* and *G. marica*: one in the Arabian Peninsula, Jordan, Syria, Oman, and Turkey (*G. marica*) and the other, larger clade from the rest of the group's Asian range east and north of the Zagros Mountains. Consistent with these results, our craniometric analysis of gazelle populations in EZ (i.e., EZIran, Caucasus, CAsia, Mongolia, and China; *n* = 61) and WZ (i.e., Arabian P, WZIran, Khark, and Borazjan *n* = 13) in relation to 14 log size and shape measurements clearly separated populations of WZ from those occurring in EZ (Figure 2). The difference between these two species may be recognized in terms of palate breadth, braincase breadth, and braincase length, and particularly size. Although size is sometimes not a good character for separating taxa, in this case, it is clear that the difference in skull size between the two species, so consistent over huge areas, can only be considered an evolutionary trait, not merely an example of phenotypic plasticity consequent on different climatic conditions.

Compared with the Arabian sand gazelle, horns average in gazelles in WZIran is slightly longer and more widely spread; braincase relatively larger both in length and width; color noticeably darker, a light brown tone, with well‐expressed face and body markings, body color between Tawny Olive and Saccardo's Umber of Ridgway (1912), mid‐face brown, somewhat darker on forehead, tending to whiten with age on nose; very thin dark brown stripes from eyes to muzzle; rest of face white. An indistinct but fairly broad stripe from elbow to stifle of hindleg, separating white of underside from brownish of body color, which just above the dark line has a broad stripe that is noticeably paler than rest of flanks. A very indistinct pygal stripe separates brown body color from white of buttocks. A fraction of females show very small horns. Gazelles in the WZIran compared with the EZIran have a narrower horn span and distance between tips of horns (Groves & Grubb, 2011), and smaller horns, braincase length, outer horn base width, palate breadth, biorbital breadth, and braincase breadth (Hemami, 1994; this study).

Some populations occurring in EZIran and CAsia are somehow separated either in size or shape from each other. According to Groves and Grubb (2011), gazelles in western Turkmenistan (*G. gracilicornis*), and those in Iran and Azerbaijan (*G. subgutturosa*) are two different phylogenetic species. Abduriyim et al. (2018) also showed that populations of *G. subgutturosa* in Turkmenistan are different from gazelles in north‐east of the Middle East and south‐east of Central Asia. Our craniometrical results confirmed that gazelles in Central Asia are different in shape from populations in EZIran and Mongolia. In addition, the results of Abduriyim et al. (2018) reported a high nucleotide divergence between southern parts of north‐western China and north‐east of the Middle East (i.e., Iran and Azerbaijan, and most of Turkmenistan) confirming the difference between gazelles of these two regions. Our morphometric analyses separated Mongolian, CAsia, and EZIran populations from each other based on shape, but the latter two showed considerable overlap in size. The implication is that Iran, CAsia, and Mongolian gazelles are craniometrically different in shape rather than size. Mongolian populations have relatively narrower tip to tip interval, palate breadth, outer horn base, and shorter horn and braincase length compared to those occurring in CAsia and EZIran. Iranian populations have the longest horns and braincase length, and CAsia populations have the widest tip to tip and outer horn base.

Cranial dimensions of Chinese gazelles, including horn length, greatest width across horn, skull length, biorbital breadth, breadth across palate, breadth of braincase, and braincase length, are greater than those from Mongolia, while the Mongolian gazelles have a larger distance between tips of horns, nasal length, and preorbital length. Most of the measured skull specimens from Mongolia (15 out of 19) were from Tsagan Nor, northern Mongolia (Table S1), in which goitered gazelle populations were extirpated about 80 years ago (Lhagvasuren, Dulamtseren, Amgalan, Mallon, & Kingswood, 2001). A large number of skull and skin specimens of these gazelles have been collected during the Asiatic Expeditions and kept at the American Museum of Natural History, New York (Allen, 1930), which could assist in clarifying the taxonomic status of this population.

Our analyses also separate the Khark Island population from other populations in size and somehow shape. A recent genetic study has suggested that the Khark Island gazelle is closer to *G. subgutturosa* than to *G. marica* (Mirzakhah et al., 2015). Nevertheless, the Khark Island population showed a strong connection with the closest population on the mainland both in geographic distance and size, that is, the presumably extinct Borazjan gazelle population named *G. karamii*, a taxon that its affiliation to *G. marica* has previously been verified (Bärmann et al., 2013). Male‐biased gene introgression between these two species (Murtskhvaladze, Gurielidze, Kopaliani, & Tarkhnishvili, 2012) could be responsible for such contradictory results. Fadakar et al. (2019) attributed these conflicting results to the “Island Rule” (Van Valen, 1973): large species show reduced body sizes on islands due to limited food resources. The origin of gazelles in Khark Island is unknown; they might represent a native or an introduced population that has been existed on this island for a long time. Further studies, primarily genetic, are required to determine the exact taxonomic status of Khark Island gazelle.

The skull originated from Borazjan district was initially described by Groves (1993) as a subspecies of *Gazella bennettii*, to which it bears little resemblance. It was transferred by Groves and Grubb (2011) to the *G. gazella* group, in which, based on the corresponding skin, it especially resembles *G. muscatensis* in its dark color and outward‐bowed horns, differing however in its white muzzle and very thin dark face stripes. The description, for the first time, of gazelles of the *G. subgutturosa/marica* group from WZIran and eastern Iraq, with their dark body color differing strongly from Arabian sand gazelle, puts this taxon into perspective. Compared to the other populations of its group, it has a smaller skull, but considerably larger horns. The color of the Khark Island gazelle population, which is the closest population to Borazjan gazelle both in geographic distance and size, is between Dresden Brown and Snuff Brown of Ridgway (1912), and so brighter than in the gazelle in south‐western Iran. It is comparatively smaller than other known populations of its group (average weight: males = 16.8 kg, *n* = 4; females: 13.2, *n* = 6; Ziaei, 1991) with noticeable flank stripes and face stripes.

### 4.2 Ecological niche divergence

Our results indicated a significant niche divergence between gazelles of EZ and WZ. This high and long mountainous belt has been acting as a serious barrier toward dispersal of species with Sahara‐Arabian origin into the central parts of Asia (Lerp, Wronski, Butynski, & Plath, 2013; Mouthereau, 2011). However, the penetration of sand gazelle's genes into EZIran has been revealed by detecting two haplotypes of *G. marica* in central and north of Iran (Fadakar et al., 2020). Our result showed that the path may have been from south‐west to south and south‐east Iran into the central and northern parts of the country. Nevertheless, the rate of gene flow from WZ to central Iran has been very low, as despite the existence of suitable habitat in central Iran for *G. marica*, no population of this species has been established. In addition, western Pakistan and south‐west of Afghnistan appeared suitable for *G. marica*; hence, the haplotypes of this species may also be detectable in these two countries as there is considerable habitat connectivity for the species in southern Iran. The same paths may have allowed the sand gazelle's ancestors to bypass the Zagros Mountains into Arabia and North Africa, and subsequently diverge into new species (Lerp et al., 2013). In EZ, a strong disconnectivity in suitable ranges of Central Asia and China was detected along the Pamir and Tian Shan Mountains. This finding implies that there might be cryptic diversification patterns in the region, which is also supported by our morphometric results.

Our habitat suitability models showed that all the studied gazelle groups are associated with arid steppes covered by consolidated and or unconsolidated lands with sparse vegetation, as also reported by Lerp et al. (2016). However, we found that they show an apparent disparity in their climatic niche space. Annual, monthly, and quarterly temperature and precipitation showed strong negative relationships with PC1. We found that along with this PC, the ecological niche of WZ gazelles (i.e., *G. marica*) is limited to warmer and drier conditions, while EZ gazelles (i.e., *G. subgutturosa*) occupy colder and more humid niche space from the west to the east.

Despite the high interspecific niche divergence, the intraspecific niche difference in *G. marica* (i.e., Arabian P and WZIran) was not significant. This is due to the uniformity of the climate and topography in the Arabian and Mesopotamian regions. Although according to the background similarity test, gazelle populations of WZIran occupy similar climatic space to the Arabian Peninsula, their habitat background is different. This may be due to the species infiltration into more temperate northern regions (near Iran and Turkey) and away from arid desert areas. Accordingly, the climatic similarity in species presence points despite the difference in their background space could be interpreted as the ecological niche conservatism (Wiens et al., 2010) in *G. marica* of the WZ.

For the gazelles in EZ (i.e., EZIran, Caucasus, CAsia, Mongolia, and China), the severity of divergence in the climatic niche was lower as in background similarity test, niche differences between gazelles of EZIran and other eastern gazelles were not significant, a pattern that was also seen clearly by a high degree of overlap in their habitat suitability. The highest niche overlap (*D* = 0.26) was seen between the gazelles of EZIran and CAsia. Although the two groups are geographically close, due to the existence of the vast Dasht‐e Kavir and Lut deserts between them and following phylogenetic findings of Fadakar et al. (2020), gazelle populations of each side were considered as a separate group.

Our results indicate that gazelle populations in EZ do not occupy significantly different climatic niches, except for those in northern Mongolian that has been adapted to cooler and more humid habitats. The lack of significant niche divergence in eastern gazelles could be a result of recent range expansion from an origin to the vacant habitats of central Asia. The Middle East is the possible origin of the genus *Gazella* from where the ancestor of goitered gazelle dispersed to the Central Asia (Lerp et al., 2016). Generally, species with greater dispersal abilities occupy larger ranges which in turn allow them better expansion into available space (Pavlicev & Mayer, 2009). Due to the high dispersal ability of large to medium‐sized species, in this case gazelles, gene flow might yet be maintaining, hampering the formation of distinct genetic structures (Ashrafzadeh, Khosravi, Ahmadi, & Kaboli, 2018), and facilitating hybridizations in contact zones (Fadakar et al., 2019). This pattern has been suggested for gazelles of Euphrates‐Tigris basin to western borders of Iran, where populations of *G. subgutturosa* and *G. marica* represent a mix of morphometric characteristics (Wacher et al., 2011). Moreover, the lack of significant topographic heterogeneity in the landscape of Central Asia might have led to a non‐significant divergence in the ecological niche.

## 5 CONCLUSION

Our morphometric and ecological niche results differentiate gazelle populations in EZ from those in WZ. Result of PCA on cranial measurements and climatic conditions placed gazelles in WZIran very close to those in the Arabian Peninsula, implying that they belong to *G. maria*, which extends the geographic range of sand gazelle to the west of the Zagros Mountains in Iran. Cryptic diversity may however exist among gazelle populations in western side of the Zagros Mountains (Fadakar et al., 2020).

Intraspecific classification of goitered gazelle has been prone to change as enough genetic, morphometric, and ecological data have not yet been available for a final conclusion. For most of the studied populations, female specimens were not available; therefore, we only used male skulls to investigate taxon differentiation. In contrast, the supportive genetic data available were primarily based on mitochondrial DNA, which is inherited maternally. We therefore incorporated additional ecological analyses (climatic niche separation and habitat suitability) to mitigate this deficiency and strengthen the morphometric results.

Nevertheless, the integration of morphometric and ecological analyses of this study with those obtained from previous genetic studies is still unconvincing and does not allow for definitive classification. The whole picture may be compromised by incomplete and opportunistic sampling, small samples sizes (particularly for our morphometric data), and the unreliability of mitochondrial markers compared to DNA fingerprinting for phylogenetic studies at the intraspecific level (Ingman, Kaessmann, Pääbo, & Gyllensten, 2000; Wan, Wu, Fujihara, & Fang, 2004). However, the notable barriers restricting gene flow among geographic population groups of goitered gazelle along with considerable geographical differentiation in skull morphology imply the existence of cryptic diversity across the species range. Therefore, based on the obtained information, albeit preliminary, we suggest splitting goitered gazelle populations into three main Management Units (MUs; Funk, McKay, Hohenlohe, & Allendorf, 2012): 1‐ EZIran MU comprising of populations between the eastern edge of Zagros Mountains and Dasht‐e Kavir/Lut Deserts, plus those in the Caucasus, 2‐ CAsia MU, including the populations in the north‐east of Iran, Turkmenistan, Afghanistan, Kazakhstan, Uzbekistan, Tajikistan, and Kyrgyzstan, and 3‐ Eastern Asia MU, comprising the rest of goitered gazelle populations occurring in Mongolia and China. More MUs may be detectable within each of these suggested main groups, as already proposed by Abduriyim et al. (2018). These results may have broad conservation implications and provide information regarding the feasibility of translocation of goitered gazelle and sand gazelle populations across much of their geographic range.

## CONFLICT OF INTEREST

The authors declare that they have no competing interests.

## AUTHOR CONTRIBUTION

**Mahmoud‐Reza Hemami:** Conceptualization (equal); Data curation (lead); Methodology (equal); Project administration (lead); Supervision (lead); Writing‐review & editing (equal). **Rasoul Khosravi:** Conceptualization (equal); Formal analysis (equal); Methodology (equal); Software (equal); Writing‐original draft (equal). **Colin Groves:** Data curation (equal); Writing‐review & editing (equal). **Mohsen Ahmadi:** Formal analysis (equal); Methodology (equal); Software (equal); Writing‐original draft (equal).

## Supporting information

Appendix S1

## Data Availability

https://doi.org/10.5061/dryad.8kprr4xkt and https://datadryad.org/stash/share/r_jjyrFx23W_Ngce4-EAFkiNSOZUlF0y33EoCbLkrnQ
