# Supplementary material for: Morphological diversity and ecological niche divergence in goitered and sand gazelles
Source: Ecol Evol. 2020 Sep 19;10(20):11535–48. doi: 10.1002/ece3.6789 (PMC7593170; doi:10.1002/ece3.6789)
Supplement: Supplementary file 1 — Appendix S1 [file ECE3-10-11535-s001.docx]

Journal of Ecology and Evolution

**Morphological diversity and ecological niche divergence in goitered and sand gazelles**

Mahmoud-Reza Hemami^1^, Rasoul Khosravi*^2^, Colin Groves ^3^, Mohsen Ahmadi^1^

**Affiliations and E-mail addresses**

^1^ Department of Natural Resources, Isfahan University of Technology, Isfahan, 8415683111, Iran

^2^ Department of Natural Resources and Environmental Engineering, School of Agriculture, Shiraz University, Shiraz 71441-65186, Iran

^2^ School of Archaeology and Anthropology, Australian National University, Canberra, ACT, Australia

**Corresponding Author**

Rasoul Khosravi Email: r-khosravi@shirazu.ac.ir

Tel: + 987136138162 Fax: + 987132276907

**Table S1** List of examined specimens. Symbols: BMNH = Natural History Museum, London; FMNH = Field Museum of Natural History, Chicago; USNM = National Museum of Natural History in Washington DC; SP = St Petersburg Museum of Natural History; AMNH = American Museum of Natural History; ZMB = Zoologisches Museum Berlin; IUT= Isfahan University of Technology, Iran; DoE = Department of Environment, Iran; MMTT = National Museum of Natural History, Iran; Moscow = Zoological Museum of Moscow University; Stockholm = Swedish Museum of Natural History; Munich = Palaeontological Museum Munich; Geneva = Natural History Museum of Geneva; Yazd DoE = Yazd Provincial Department of Environment, Iran; Yazd NHM = Natural History Museum of Yazd, Iran; Isfahan DoE = Isfahan Provincial Department of Environment, Iran; Kolah Qazi = Kolah Qazi National Park, Iran.

|  | Place of storage | label | locality |  | Place of storage | label | locality |
| --- | --- | --- | --- | --- | --- | --- | --- |
| 1 | Moscow | S-38871 | Badkhyz- Turkmenistan | 38 | Yazd DoE | no number | Yazd- Iran |
| 2 | Moscow | S-51740 | South of Turkmenistan | 39 | Yazd NHM | no number | Yazd- Iran |
| 3 | Moscow | S-51744 | South of Turkmenistan | 40 | AMNH | 57261 | Tsagan Nor- Mongolia |
| 4 | Moscow | S-53362 | Kyzyl-Kum- Turkmenistan | 41 | AMNH | 57257 | Tsagan Nor- Mongolia |
| 5 | Moscow | S-51753 | Kushke –Turkmenia | 42 | AMNH | 57265 | Tsagan Nor- Mongolia |
| 6 | Moscow | S-38885 | Gyaurs- Turkmenistan | 43 | AMNH | 57255 | Tsagan Nor- Mongolia |
| 7 | Moscow | S-51754 | Kushke - Turkmenistan | 44 | AMNH | 57258 | Tsagan Nor- Mongolia |
| 8 | Moscow | S-38893 | Gyaurs- Turkmenistan | 45 | AMNH | 57270 | Tsagan Nor- Mongolia |
| 9 | Moscow | S-38892 | Gyaurs- Turkmenistan | 46 | AMNH | 57272 | Tsagan Nor- Mongolia |
| 10 | Moscow | S-38905 | Badkhyz- Turkmenistan | 47 | AMNH | 57267 | Tsagan Nor- Mongolia |
| 11 | Moscow | S-38869 | Saradzhe- Turkmenistan | 48 | AMNH | 54915 | Kashgarian plains- China |
| 12 | SP Petersburg | 8400 | Bey-Shan- China | 49 | AMNH | 97792 | Bey-Shan- China |
| 13 | SP | 7838 | Tsaidam –China | 50 | BMNH | 91.8.7.91 | Yarkand- China |
| 14 | SP | 8408 | Karadun Hills- Uzbekistan | 51 | BMNH | 91.8.7.88 | Yarkand- China |
| 15 | SP | 8382 | Semiretsch- Kyrgyzstan | 52 | Stockholm | 4/1148 | Pangchiang- Mongolia |
| 16 | SP | 8405 | Bey-Shan- China | 53 | Stockholm | no number | Tehran- Iran |
| 17 | SP | 8407 | Karadun Hills- Uzbekistan | 54 | Munich | 1957.264 | Khuzestan- Iran |
| 18 | SP | 8379 | Gobi- China | 55 | Geneva | 689/13 | Caucasus |
| 19 | SP | 7775 | Gobi- China | 56 | Taif | no number | Harrat al Arrat -Jordan |
| 20 | SP | 18435 | Tashkepri- Turkmenistan | 57 | BMNH | 48.397 | Es Taisiyah Nejd- Arabia |
| 21 | SP | 8409 | Ulyasutai- Mongolia | 58 | BMNH | 48.396 | Es Taisiyah Nejd- Arabia |
| 22 | BMNH | 47.74 | Isfahan- Iran | 59 | BMNH | 48.393 | North Arabia |
| 23 | BMNH | 23.10.18.1 | Mahammerah- Iran | 60 | BMNH | 39.553 | Palmyra- Syria |
| 24 | FMNH | 26680 | Loh- Mongolia | 61 | MMTT | 402 | Khosh Yeylagh- Iran |
| 25 | USNM | 240691 | Paotow- Mongolia | 62 | MMTT | 624 | Khosh Yeylagh- Iran |
| 26 | AMNH | 57241 | Tsagan Nor- Mongolia | 63 | MMTT | 625 | Khosh Yeylagh- Iran |
| 27 | AMNH | 57234 | Tsagan Nor-Mongolia | 64 | MMTT | 677 | Khosh Yeylagh- Iran |
| 28 | Greg Harrison | 1.5725 | Ramlat Fasd-Arabia | 65 | Isfahan DoE | no number | Mooteh- Iran |
| 29 | AMNH | 57242 | Tsagan Nor- Mongolia | 66 | MMTT | 616 | Near Mooteh- Iran |
| 30 | AMNH | 57236 | Tsagan Nor- Mongolia | 67 | MMTT | 614 | Mooteh-- Iran |
| 31 | AMNH | 57248 | Tsagan Nor- Mongolia | 68 | MMTT | B-2-A/MO/7 | Mooteh- Iran |
| 32 | AMNH | 57250 | Tsagan Nor- Mongolia | 69 | MMTT | 613 | Mooteh- Iran |
| 33 | AMNH | 57249 | Tsagan Nor- Mongolia | 70 | Trophy hunting | no number | Kolah Qazi- Iran |
| 34 | IUT | 001 | Khark- Iran | 71 | Kolah Qazi | no number | Kolah Qazi- Iran |
| 35 | IUT | 002 | Khark- Iran | 72 | Kolah Qazi | no number | Kolah Qazi- Iran |
| 36 | IUT | 003 | Khark- Iran | 73 | MMTT | 416 | Dashte-Zahab- Iran |
| 37 | Yazd DoE | no number | Yazd- Iran | 74 | ZMB | 41400 | Borazjan, Bushehr, Iran |

**Table S2**. *G. subgutturosa* group skull and horn measures and abbreviations

|  | Measurement | Abbreviation |  | Measurement | Abbreviation |
| --- | --- | --- | --- | --- | --- |
| 1 | Horn Length | HL | 8 | Skull length | SL |
| 2 | Distance between tips of horns | DBTH | 9 | Biorbital breadth | BB |
| 3 | Greatest width across horns | GWH | 10 | Preorbital length | PL |
| 4 | Outer horn base width | OHW | 11 | Maxillary tooth row Length | MTRL |
| 5 | Breadth across both nasals, anterior | BNA | 12 | Breadth across palate | PB |
| 6 | Breadth across both nasals, posterior | BNP | 13 | Breadth of brain case | BBC |
| 7 | Nasal length | NL | 14 | Braincase length | BL |

**Table S3**. The number of gazelles’ presence points collected for performing ENM analysis.

| Geographic group | Number of presence points |
| --- | --- |
| Arabian P | 62 |
| WZIran | 11 |
| EZIran | 106 |
| Caucasus | 9 |
| Turkmenistan | 18 |
| China | 50 |
| Mongolia | 20 |
| Total | 280 |

**Table S4**. Basic statistics (in mm) of 14 skull measurements (see table S2 for the explanation of abbreviations). The sample size for each location is shown in parenthesis

|  | Arabian P  (6) | | WZIran  (3) | | Khark  (3) | | EZIran  (17) | | Caucasus | CAsia  (14) | | Mongolia  (17) | | China  (12) | | Borazjan (1) |
| --- | --- | --- | --- | --- | --- | --- | --- | --- | --- | --- | --- | --- | --- | --- | --- | --- |
|  | *X̅* | CV* | *X̅* | CV* | *X̅* | CV* | *X̅* | CV* |  | *X̅* | CV* | *X̅* | CV* | *X̅* | CV* |  |
| HL | 272.33 | 9.79 | 286 | 14.39 | 314 | 8.08 | 331.29 | 8.98 | 282 | 309.05 | 9.37 | 254.76 | 5.51 | 279.44 | 9.41 | 228 |
| DBTH | 101.83 | 51.23 | 155.93 | 32.14 | 113.9 | 31.29 | 136.86 | 42.02 | 107 | 153.21 | 34.54 | 113 | 33.34 | 98.67 | 32.72 | 190 |
| GWH | 171.5 | 26.82 | 203 | 17.93 | 200.06 | 34.35 | 199.67 | 21.33 | 196 | 225.37 | 16.71 | 166.24 | 17.31 | 171.83 | 13.62 | 210 |
| OHW | 56.83 | 5.35 | 61.53 | 3.26 | 60.83 | 2.24 | 66.13 | 5.24 | 68 | 74.8 | 2.26 | 63.24 | 3.97 | 72.16 | 8.66 | 58 |
| BNA | 24.5 | 10.31 | 24.2 | 4.72 | 23.1 | 2.03 | 22.57 | 5.13 | 26 | 25.66 | 5.66 | 26.94 | 4.86 | 27.28 | 7.15 | 21 |
| BNP | 23.66 | 10.64 | 23.83 | 9.15 | 21.03 | 0.78 | 23.91 | 9.9 | 27 | 27.27 | 5.09 | 28.97 | 5.45 | 29.02 | 7.58 | 22 |
| NL | 48.58 | 5.34 | 47.2 | 16.03 | 41.86 | 12.21 | 58.24 | 14.13 | 60 | 56.11 | 10.66 | 60.53 | 7.67 | 58.93 | 9.56 | 41 |
| SL | 184.83 | 3.11 | 186.83 | 2.92 | 173.83 | 3.45 | 210.15 | 4.15 | 219 | 204.47 | 1.98 | 204.94 | 2.31 | 209.38 | 2.67 | 173 |
| BB | 84.41 | 2.8 | 89.36 | 1.43 | 88.36 | 0.14 | 96.03 | 2.73 | 101 | 96.12 | 2.15 | 95.18 | 2.16 | 96.8 | 2.74 | 84 |
| PL | 94.5 | 2.48 | 94.73 | 5.94 | 86.96 | 6.78 | 104.72 | 24.29 | 117 | 39.17 | 13.19 | 107.41 | 3.53 | 69.51 | 53.36 | 87 |
| MTRL | 56.16 | 1.82 | 57.1 | 3.45 | 53.96 | 1.47 | 63.17 | 2.68 | 65 | 63.85 | 4.3 | 61.18 | 4.33 | 66.66 | 4.55 | 56 |
| PB | 55 | 4.31 | 52.83 | 2.12 | 52.5 | 2.05 | 59.93 | 8.08 | 62 | 57.5 | 4.46 | 59.65 | 2.86 | 59.62 | 2.59 | 49 |
| BBC | 66.25 | 5.43 | 69.23 | 3.67 | 59.4 | 3.62 | 72.07 | 4.58 | 72 | 71.98 | 4.14 | 71.35 | 4.79 | 73.99 | 3.23 | 58 |
| BL | 103.33 | 3.22 | 109.5 | 4.7 | 107.33 | 0.95 | 116.28 | 6.15 | 119 | 112.14 | 3.15 | 106.76 | 2.74 | 102.72 | 28.52 | 98 |

**Table S5** Summary of the principal components analysis on the 19 bioclimatic variables extracted from the area defined by a 50-km buffer around gazelles presence points.

| Climatic variables | PC1 | PC2 | PC3 | PC 4 |
| --- | --- | --- | --- | --- |
| BIO1 annual mean temperature (°C) | -0.97 | -0.05 | -0.05 | -0.15 |
| BIO2 mean diurnal range (°C) | -0.92 | 0.05 | -0.04 | -0.15 |
| BIO3 isothermality | -0.98 | -0.14 | -0.05 | -0.12 |
| BIO4 temperature seasonality | 0.35 | -0.89 | 0.23 | -0.07 |
| BIO5 max. temperature of the warmest month (°C) | 0.48 | -0.7 | 0.45 | -0.24 |
| BIO6 min. temperature of the coldest month (°C) | 0.41 | -0.55 | -0.63 | 0.03 |
| BIO7 temperature annual range (°C) | 0.13 | 0.43 | 0.65 | -0.48 |
| BIO8 mean temperature of the wettest quarter (°C) | 0.45 | -0.64 | 0.44 | -0.2 |
| BIO9 mean temperature of the driest quarter (°C) | 0.41 | -0.58 | -0.63 | 0.04 |
| BIO10 mean temperature of the warmest quarter (°C) | 0.89 | -0.04 | 0.09 | -0.28 |
| BIO11 mean temperature of the coldest quarter (°C) | -0.37 | -0.56 | 0.36 | 0.07 |
| BIO12 annual precipitation (mm) | -0.2 | 0.32 | 0.62 | 0.51 |
| BIO13 precipitation of the wettest month (mm) | -0.84 | -0.07 | 0.31 | 0.05 |
| BIO14 precipitation of the driest month (mm) | 0.86 | 0.31 | 0.04 | 0.05 |
| BIO15 precipitation seasonality | -0.92 | 0.01 | 0.04 | -0.03 |
| BIO16 precipitation of the wettest quarter (mm) | -0.97 | -0.19 | -0.07 | -0.14 |
| BIO17 precipitation of the driest quarter (mm) | 0.79 | 0.37 | 0.18 | 0.23 |
| BIO18 precipitation of the warmest quarter (mm) | 0.03 | 0.57 | -0.26 | -0.64 |
| BIO19 precipitation of the coldest quarter (mm) | -0.94 | -0.27 | -0.03 | 0.11 |
| Eigenvalue | 9.34 | 4.03 | 2.41 | 1.24 |
| Explained variance (%) | 49.61 | 20.82 | 12.71 | 6.54 |


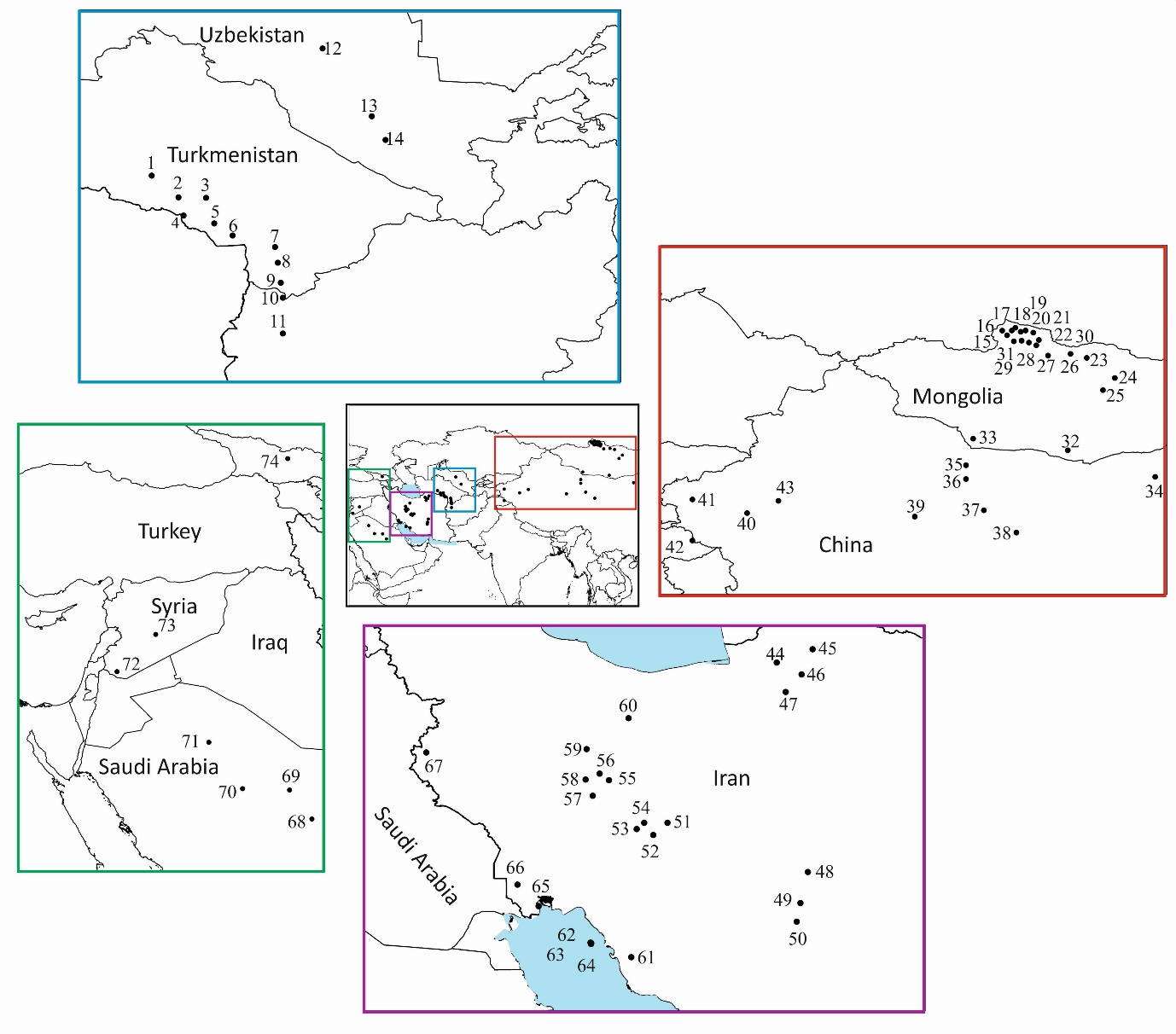
**Fig. S1**. Locations where gazelle skulls were collected (black points). See table S1 for geographic names and locality abbreviations.


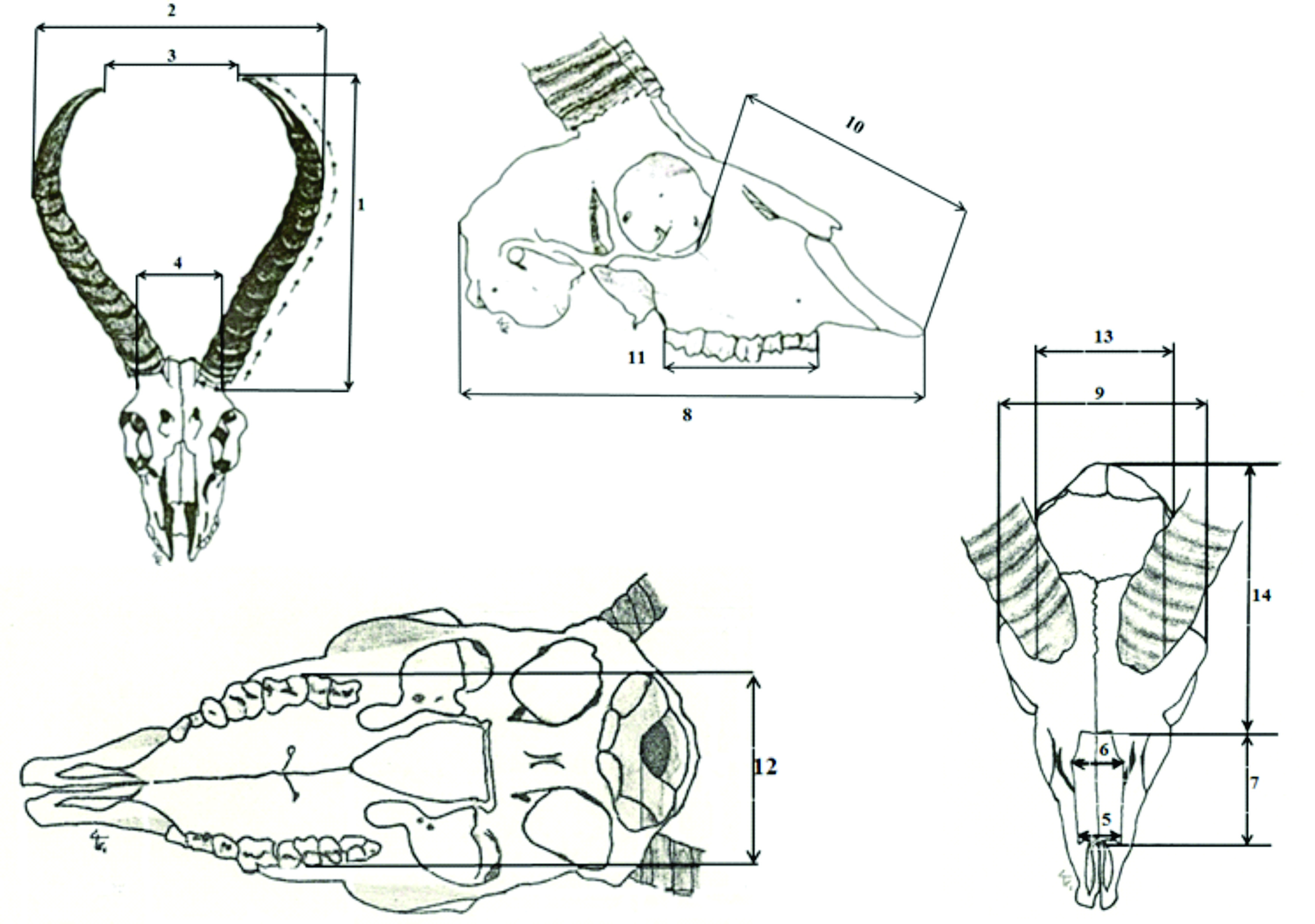


**Fig. S2.** Morphological measurements of Persian gazelles (Reprinted from Hemami, 1994).


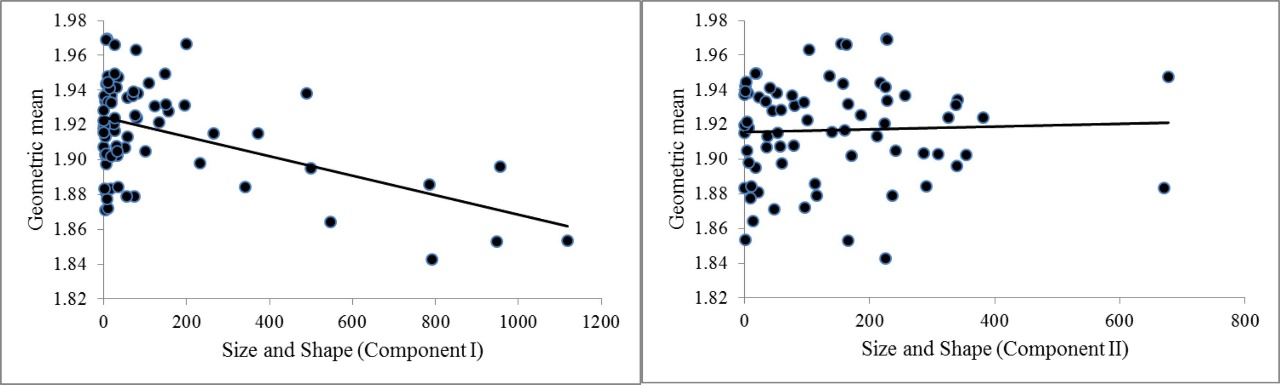


**Fig. S3.** The scores of the individual measures plotted the respective geometric means on the first log size and shape components (left; R^2^= 0.23) and on the second (right; R^2^= 0.00).


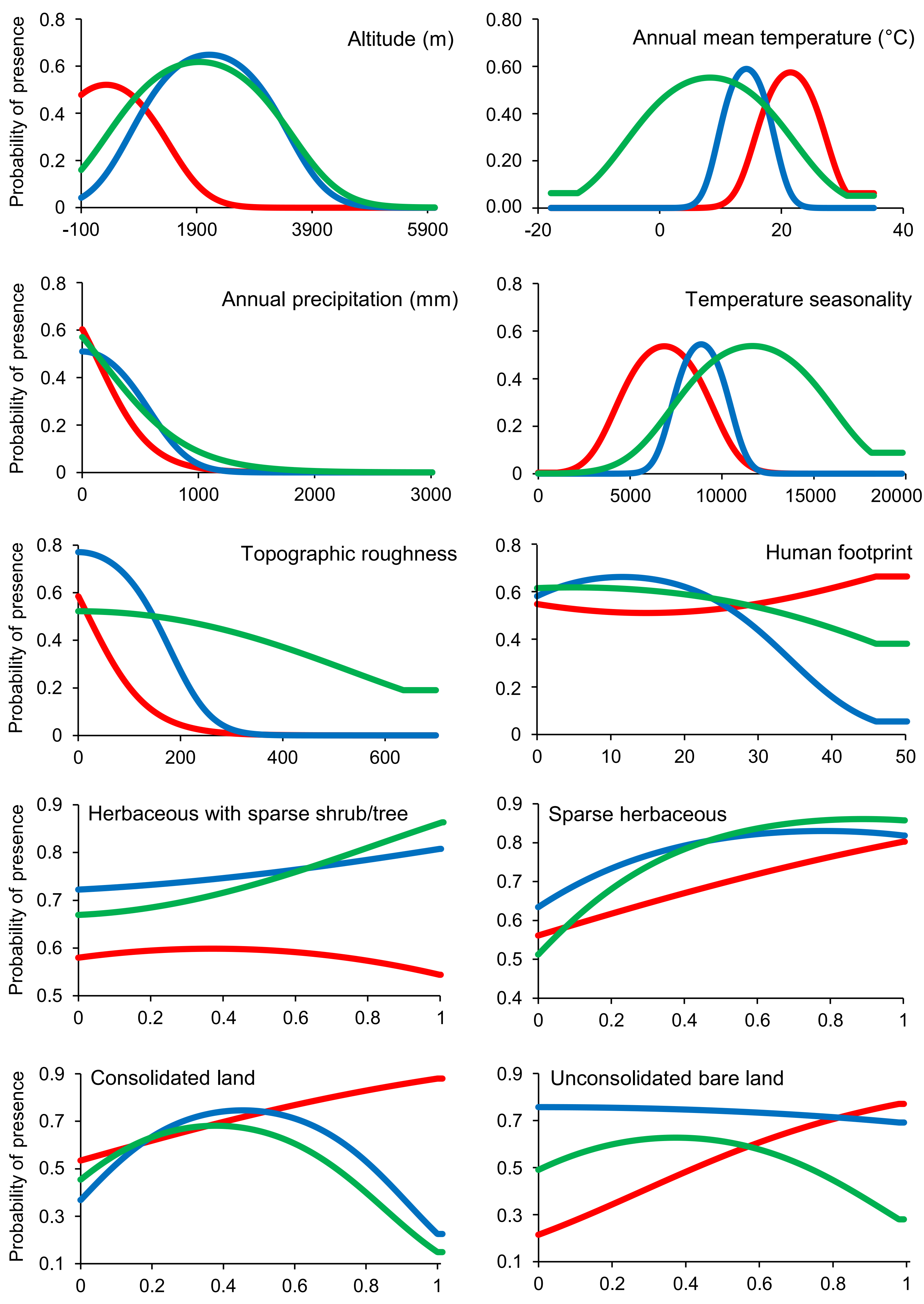


**Fig. S4.** The response curve of the environmental variables affecting habitat suitability of *G. marica* (red), *G. subgutturosa subgutturosa* (green), and *G. s. yarkandensis* (blue) derived based on the MaxEnt model.
